# Full Reperfusion Without Functional Independence After Mechanical Thrombectomy in the Anterior Circulation: Performance of Prediction Models Before Versus After Treatment Initiation

**DOI:** 10.1007/s00062-022-01166-x

**Published:** 2022-05-09

**Authors:** Charlotte S. Weyland, Johannes A. Vey, Yahia Mokli, Manuel Feisst, Meinhard Kieser, Christian Herweh, Silvia Schönenberge, Markus A. Möhlenbruch, Martin Bendszus, Peter A. Ringleb, Simon Nagel

**Affiliations:** 1 Department of Neuroradiology, Heidelberg University Hospital, Heidelberg, Germany; 2 Institute of Medical Biometry, Heidelberg University Hospital, Heidelberg, Germany; 3 Department of Neurology, Heidelberg University Hospital, Im Neuenheimer Feld 400, 69120 Heidelberg, Germany; 4 Department of Psychiatry and Psychotherapy, Giessen and Marburg University Hospital, Marburg, Germany

**Keywords:** Futile recanalization, Ischemic stroke, Outcome prediction, Logistic regression models, Early neurological improvement

## Abstract

**Background and Purpose:**

Prediction of futile recanalization (FR), i.e. failure of long-term functional independence despite full reperfusion in mechanical thrombectomy (MT), is instrumental in patients undergoing endovascular therapy.

**Methods:**

Retrospective single-center analysis of patients treated for anterior circulation LVO ensuing successful MT (mTICI 2c–3) between January 2014 and April 2019. FR was defined as modified Rankin Scale (mRS) 90 days after stroke onset > 2 or mRS > pre-stroke mRS. Multivariable analysis was performed with variables available before treatment initiation regarding their association with FR. Performance of the regression model was then compared with a model including parameters available after MT.

**Results:**

Successful MT was experienced by 549/1146 patients in total. FR occurred in 262/549 (47.7%) patients. Independent predictors of FR were male sex, odds ratio (OR) with 95% confidence interval (CI) 1.98 (1.31–3.05, *p* 0.001), age (OR 1.05, CI 1.03–1.07, *p* < 0.001), NIHSS on admission (OR 1.10, CI 1.06–1.13, *p* < 0.001), pre-stroke mRS (OR 1.22, CI 1.03–1.46, *p* 0.025), neutrophile-lymphocyte ratio (OR 1.03, CI 1.00–1.06, *p* 0.022), baseline ASPECTS (OR 0.77, CI 0.68–0.88, *p* < 0.001), and absence of bridging i.v. lysis (OR 1.62, 1.09–2.42, *p* 0.016). The prediction model’s Area Under the Curve was 0.78 (CI 0.74–0.82) and increased with parameters available after MT to 0.86 (CI 0.83–0.89) with failure of early neurological improvement being the most important predictor of FR (OR 15.0, CI 7.2–33.8).

**Conclusion:**

A variety of preinterventional factors may predict FR with substantial certainty, but the prediction model can still be improved by considering parameters only available after MT, in particular early neurological improvement.

**Supplementary Information:**

The online version of this article (10.1007/s00062-022-01166-x) contains supplementary material, which is available to authorized users.

## Introduction

Mechanical thrombectomy (MT) of large vessel occlusion (LVO) in the anterior cerebral circulation for patients with acute ischemic stroke has become standard of care in recent years [1]. With an ever-growing number of patients being treated with MT worldwide over the last years, a broad range of factors predicting good clinical outcome after MT were described. Based on these, a plethora of outcome prediction models were developed with different purposes and variable settings [2]. The majority of prediction models focus on good clinical outcome after treatment measured by the modified Rankin Scale (mRS), which consists of seven levels between no clinical deficit (mRS = 0) and death (mRS = 6) [3]. Allowing these prediction models access to everyday stroke care and decision making is arguably difficult. For optimal patient care, however, predicting an unfavorable outcome despite (assumed) technical successful MT procedure may be at least as relevant.

Futile recanalization (FR) refers to patients, who do not experience a good clinical long-term outcome despite successful recanalization. The definition of good clinical outcome varies as well and was defined as functional independence for this study. The phenomenon of FR is not uncommon with reported rates of up to 30–50% in modern stroke centers [4]. Variables associated with FR can be clinical, such as initial NIHSS [5], laboratory parameters like blood glucose level on admission [4] and baseline imaging parameters (e.g., extent of the cerebral infarction [6], degree of pre-existing cerebral microangiopathy [7]) as well as interventional parameters, such as the number of thrombectomy attempts [8]. Infarct locations currently discussed to be associated with FR are the central motor region (ASPECTS region M4), the parietal cortex (ASPECTS region M6) and the basal ganglia to a varying extent in different studies [9].

A very strong predictor for FR, which is unknown for the individual patient until after MT, is failure of early neurological improvement (ENI) after mechanical thrombectomy [10, 11]. To assess predictors of FR after acute ischemic stroke of the anterior circulation, we firstly included variables available before treatment decision in a logistic regression model. This was then compared to a prediction model including also variables, that are available after MT. An example for a variable known only after MT is early neurological improvement. The information about the latter would not be available before physicians need to decide whether to treat a patient or not during acute stroke treatment.

## Methods

This study was approved by the local ethics committee and has therefore been performed in accordance with the ethical standards laid down in the 1964 Declaration of Helsinki and its later amendments.

This retrospective single-center analysis includes prospectively treated patients with LVO in the anterior circulation within a 5-year study period (January 2014 until April 2019). Only patients with a successful MT were included. To avoid a bias concerning the quality of target vessel recanalization, successful MT was defined as modified thrombolysis in cerebral infarction (mTICI) score 2c or 3. Patients with an incomplete final MT result of mTICI 2b or less were excluded.

The clinical decision for treating patients with MT was made by an experienced neuroradiologist in consensus with an experienced neurologist and was based on local standard operating procedures as well as national guidelines. During April 2014 and February 2016, the mode of sedation (conscious sedation vs. general anesthesia) was randomized within the SIESTA trial [12]. Imaging protocol included at least either CT with CT-angiography or MRI with MR-angiography. Within the local stroke database all clinical parameters were obtained by NIHSS/mRS-certified neurologists. ASPECTS was automatically calculated by e‑ASPECTS on baseline scans and as well as follow-up scans, additionally scored or verified by an experienced neuroradiologist (4 years radiological experience) [13]. The load of microangiopathic cerebral lesions according to the Swieten scale [14] and collateral status according to the Tan score [15] on baseline images were manually evaluated. The clinical outcome as per mRS 90 days after stroke onset was obtained by a standardized interview (unblinded investigator per telephone call or a personal letter to the patient). The recanalization result according to the mTICI score was determined by the interventional neuroradiologist in charge of the MT.

### Primary Endpoint and Included Variables

The primary endpoint of the study was a modified Rankin Scale (mRS) score of 3–6, 3 months after stroke onset or worse than baseline if the premorbid mRS was ≥ 3. Patients who experienced FR were compared to patients with substantial clinical improvement after MT (mRS 90 days after stroke onset ≤ 2 or equal to pre-stroke mRS). Overall, 38 clinical, radiological, and laboratory variables were tested for association with FR. Laboratory variables were collected retrospectively from the electronic patient charts.

### Statistical Analysis

The patient cohort was described using summary measures of the empirical distribution and potential risk factors for the primary endpoint were investigated using univariate analyses. Nominal variables are given as absolute and relative frequencies and continuous variables are reported as mean ± standard deviation (SD) or median and interquartile range, as appropriate. The t‑test for independent groups or the Mann-Whitney-Wilcoxon test, as appropriate, were used. For categorical variables the χ^2^-test and for dichotomous variables the Fisher-Boschloo test were applied.

Missing values were multiply imputed for multivariable analysis. To select variables with predictive impact on the primary endpoint regularized logistic regression using the elastic net penalty was applied. The hyperparameters α and λ were tuned by grid search and 5‑fold cross-validation (CV) [16]. Subsequently, multivariable regression modeling was conducted based on the 10 imputed data sets only incorporating the selected variables. In order to produce unbiased and finite parameter estimates, Firth’s bias-reduced penalized likelihood logistic regression [17] was applied and pooled confidence intervals of the parameter estimates were computed conducting the combination of penalized likelihood profiles (CLIP) method [18]. The discriminatory performance of the final model was assessed using 5‑fold CV by applying the receiver operating characteristic (ROC) and computing the area under curve (AUC) with its corresponding 95% confidence interval (CI) according to DeLong et al. [19]. The model calibration was assessed by calculating the calibration intercept and slope [20]. For the development of the two prediction models, the same methodical strategy was applied but a different set of variables was used as initial candidate predictors (variables available before and after MT, see Table 1). The models are reported according to the TRIPOD statement [21]. Details of the model development are described in the supplement.

**Table 1:** Parameters associated with futile recanalization (FR) after successful mechanical thrombectomy (MT)

Variables	Successful MT, clinically not futile 287 (549)	FR (262/549)	*p*-value
**Baseline clinical parameters**			
*Age, mean (SD)*	71 (13)	78 (11)	**<** **0.001**
*Sex, male, n (%)*	142 (49)	131 (50)	0.905
*Pre-stroke mRS, median (min–max, interquartile range)*	0 (0–2)	2 (0–3)	**<** **0.001**
*Initial NIHSS score, median (min–max)*	13 (0–41)	18 (1–38)	**<** **0.001**
*Wake-up stroke (unknown onset time), n (%)*	60 (21)	66 (25)	0.242
**Comorbidities**			
Arterial hypertension, *n* (%)	222 (77)	218 (83)	0.092
Diabetes mellitus, *n* (%)	59 (21)	70 (27)	0.102
Hypercholesterolemia, *n* (%)	103 (36)	95 (37)	0.901
Active smoking, *n* (%)	42 (15)	31 (12)	0.370
Previous stroke, *n* (%)	44 (15)	68 (26)	**0.002**
Coronary heart disease, *n* (%)	71 (25)	85 (32)	**0.049**
Peripheral artery disease (PAD), *n* (%)	17 (6)	24 (9)	0.166
End stage renal failure (dialysis patient), *n* (%)	0 (0)	8 (3)	**0.004**
Atrial fibrillation, *n* (%)	137 (48)	154 (59)	**0.008**
**Blood levels on admission**			
Glucose (mg/dl), mean (SD)	125 (40)	113 (83)	**<** **0.001**
HbA1c [%], mean (SD)	6.0 (30.9)	6.3 (1.6)	**0.008**
Neutrophil-lymphocyte ratio, mean (SD)	7.5 (8.5)	10.0 (10.5)	**0.012**
**Imaging aspects before treatment**			
ASPECTS on admission, median (IQR)	9 (8–10)	9 (7–10)	**<** **0.001**
Collateral score (Tan), median (IQR)	3 (2–4)	3 (2–3)	**<** **0.001**
*Cerebral micoangiopathy score (Swieten), n (%)*			
1	204 (73%)	130 (52%)	**<** **0.001**
2	47 (17%)	73 (29%)	
3	27 (10%)	45 (18%)	
*Target vessel occlusion side*			
Distal ICA, *n* (%)	20 (7)	12 (5)	**0.045**
ICA and MCA, *n* (%)	24 (8)	19 (7)	
Carotid‑T, *n* (%)	45 (16)	62 (24)	
MCA, M1-segment, *n* (%)	138 (48)	132 (50)	
MCA, M2-segment, *n* (%)	60 (21)	37 (14)	
**Treatment aspects**			
MT only, no intravenous thrombolysis, *n* (%)	110 (38)	132 (50)	**0.005**
Number of thrombectomy attempts, *median (IQR)*	1 (1–2)	1.5 (1–3)	**<** **0.001**
General anesthesia during MT, *n* (%)	50 (17)	70 (27)	**0.009**
Stenting of extracranial ICA during MT, *n* (%)	38 (14)	35 (14)	0.916
Time from onset to initial treatment (min), mean (SD)	173 (145)	177 (115)	0.765
Time from hospital admission to groin puncture (min), mean (SD)	84 (67)	76 (35)	0.073
Time from groin puncture to final recanalization result (min), mean (SD)	98 (74)	105 (57)	0.222
*Target vessel recanalization result (according to mTICI)*			
mTICI 2c	66 (23)	71 (27)	0.274
mTICI 3	221 (77)	191 (73)	
**Relevant variables known after initial therapy**			
Failure of early neurological improvement, *n* (%)	13 (5)	102 (39)	**<** **0.001**
ASPECTS follow-up, median (IQR)	8 (7–10)	7 (4–8)	**<** **0.001**
Intracranial hemorrhage, asymptomatic	38 (13)	57 (22)	**0.009**
ICH symptomatic	3 (1)	14 (5)	**0.005**

*MT* mechanical thrombectomy, *FR* futile recanalization, *mRS* modified Rankin Scale, *NIHSS* National Institutes of Health Stroke Scale, *HbA1c* Hemoglobin A1c, *ASPECTS* Alberta Stroke Program Early CT Score, *ICA* Internal Carotid Artery, *MCA* Middle Cerebral Artery, *mTICI* modified Treatment In Cerebral Ischemia, *ICH* IntraCranial Hemorrhage, *IQR* InterQuartile Range, *SD* Standard Deviation

Furthermore, individual ASPECTS regions in follow-up imaging (CT 24 h after intervention) were analyzed performing univariate and multivariable logistic regression modeling to assess their association with FR.

Since this was a retrospective data analysis, all *p* values have to be interpreted in a descriptive sense; *p* values < 0.05 were denoted as significant. All statistical analyses were performed using the software R version ≥ 4.0.3 (www.r-project.org).

## Results

In the study period 1146 patients were treated with MT for a large vessel occlusion of the anterior circulation. The analysis included 549 patients (47.9%) who had an excellent technical recanalization result (mTICI 2c/3) and 3months after stroke onset 262/549 patients (47.7%) showed a FR. In univariate analysis, a set of variables were different regarding the two study groups (see Table 1). Patients with FR were older (age in years as mean; standard deviation: 78; 11 FR vs. 71; 13 no-FR, *p* < 0.001), had a higher NIHSS on admission (mean; SD: 18; 6.6 FR vs. 13; 6.8 no-FR, *p* < 0.001) and a higher pre-stroke mRS (median, IQR: 2; 0–3 FR vs. 0; 0–2 no-FR, *p* < 0.001). Concerning vascular risk factors, patients with FR were more likely to have had a previous stroke (*n*; %: 68; 26% FR vs. 44; 15% no-FR, *p* = 0.002), had a higher rate of known atrial fibrillation (*n*; %: 154; 59% FR vs. 137, 48% no-FR, *p* = 0.008) as well as a higher blood glucose level on admission (mean; SD in mg/dl: 137; 62 FR vs. 125; 43 no-FR, *p* = 0.007). With respect to preinterventional imaging, patients with FR showed a lower ASPECTS on admission (median; IQR: 9; 7–10 FR vs. 9; 8–10 no-FR, *p* < 0.001), a lower collateral score (Tan score median; IQR: 3; 2–3 FR vs. 3; 2–4 no-FR, *p* < 0.001), and a higher cerebral microangiopathy score (Swieten score as median; IQR: 1; 1–2 FR vs. 1; 1–2 no-FR, *p* < 0.001). Regarding treatment aspects, patients with FR were more likely to have had no bridging i.v. thrombolysis (*n*; %: 132; 50% FR vs. 110; 38% no-FR, *p* = 0.005), a higher number of thrombectomy attempts (median; IQR: 1.5; 1–3 FR vs. 1; 1–2 no-FR, *p* < 0.001) and were more often treated with the patient under general anesthesia (*n*; %: 70; 27% FR vs. 50; 17% no-FR, *p* = 0.009). Regarding factors, that were known after initial treatment, patients with FR more often showed also no early neurological improvement (*n*; %: 102; 39% FR vs. 13; 5% no-FR, *p* < 0.001) and had a lower ASPECTS in follow-up imaging (median; IQR: 7; 5–8 FR vs. 9; 8–10 no-FR, *p* < 0.001). Moreover, patients with FR also more frequently showed asymptomatic intracranial hemorrhage (*n*; % 57 22% FR vs. 38; 13% no-FR, *p* = 0.009) as well as symptomatic intracranial hemorrhage (*n*; %: 14; 5% FR vs. 3; 1% no-FR, *p* = 0.005).

## Multivariable Analysis

**Fig. 1:**
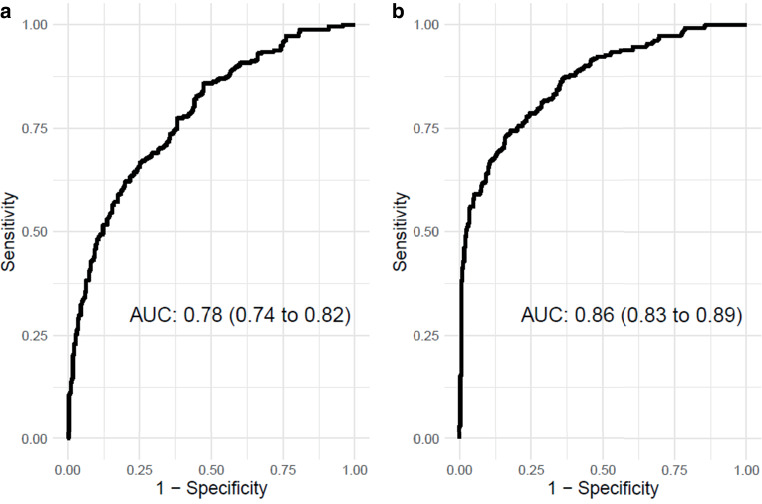
Receiver operating characteristic (ROC) curves for the prediction of futile recanalization after acute ischemic stroke considering only variables known before mechanical thrombectomy (**a**) and variables known after mechanical thrombectomy (**b**)

When considering only variables known before initial treatment for variable selection, independent predictors for FR in the final model were male sex (OR (CI) 1.98 (1.31–3.05), *p* = 0.001), absence of thrombolysis before MT (1.62 (1.09–2.42), *p* = 0.016), pre-stroke mRS (OR (CI): 1.22 (1.03–1.46, *p* = 0.025)), NIHSS on admission (OR (CI) 1.10 (1.06–1.13), *p* < 0.001), age (OR (CI) 1.05 (1.03–1.07), *p* < 0.001), NL Ratio (OR (CI) 1.03 (1.00–1.06), *p* = 0.022) and ASPECTS on admission (OR (CI) 0.77 (0.68–0.88), *p* < 0.001), (see Table 2). The ROC analysis of these variables showed an AUC (CI) of 0.78 (0.74–0.8) (see Fig. 1). Assessing the calibration of the model revealed very good calibration with calibration intercept of 0.01 and calibration slope of 0.93 (see supplemental material, figure I).

**Table 2:** Final logistic prediction model for futile recanalization based on variables known before initial treatment (with intercept = −4.015)

Parameters	*p*-value	Odds ratio (CI)
**Age**	**<** **0.001**	1.047 (1.027–1.069)
**Sex (male)**	**0.001**	1.982 (1.305–3.045)
**NIHSS on admission**	**<** **0.001**	1.098 (1.064–1.134)
**MT only, no intravenous thrombolysis**	**0.016**	1.622 (1.093–2.418)
**Pre-stroke mRS**	**0.025**	1.221 (1.026–1.456)
**Neutrophil/Lymphocyte ratio**	**0.022**	1.032 (1.004–1.064)
**ASPECTS on admission**	**<** **0.001**	0.774 (0.678–0.879)
**Swieten (reference: Swieten scale 1)**		
Swieten scale 2	0.082	1.552 (0.945–2.556)
Swieten scale 3	0.718	1.120 (0.605–2.081)
*End stage renal failure (dialysis patient)*	0.158	9.593 (0.415–221.5)

Bold type was used for statistical significant values

*NIHSS* National Institutes of Health Stroke Scale, *MT* mechanical thrombectomy, *i.v.* intravenous, *mRS* modified Rankin Scale, *NL-ratio* neutrophil-lymphocyte ratio in peripheral blood, *ASPECTS* Alberta Stroke Program Early CT Score, *CI* 95% Confidence Interval

When developing the model considering also variables only recordable after thrombectomy, failure of early neurological improvement (OR (CI) 14.97 (7.22–33.793, *p* < 0.001)), lower ASPECTS in follow-up imaging (OR (CI) 0.63 (0.55–0.72), *p* < 0.001), were additional independent predictors for FR in the final model (see Table 3). The according ROC analysis of the model showed a better discriminatory power (AUC of 0.86 (CI 0.83–0.89)) and very good calibration (calibration intercept of −0.02 and calibration slope of 0.93, Fig. 1 and supplemental material, figure II).

**Table 3:** Final logistic prediction model for futile recanalization based on variables known before and after initial treatment with mechanical thrombectomy (with intercept = −3.071)

Parameters	*p*-value	Odds ratio (CI)
**Failure of early neurological improvement**	**<** **0.001**	14.966 (7.220–33.793)
**Age**	**<** **0.001**	1.053 (1.029–1.078)
**NIHSS on admission**	**<** **0.001**	1.084 (1.047–1.125)
*MT only, no intravenous thrombolysis*	0.081	1.498 (0.951–2.367)
**Pre-stroke mRS**	**0.011**	1.294 (1.061–1.581)
*Neutrophil/Lymphocyte ratio*	0.069	1.029 (0.998–1.063)
**ASPECTS follow-up**	**<** **0.001**	0.631 (0.546–0.720)
**Swieten (reference: Swieten scale 1)**	**–**	–
Swieten scale 2	**0.018**	1.985 (1.126–3.518)
Swieten scale 3	0.654	0.841 (0.392–1.783)

Bold type was used for statistical significant values

*NIHSS* National Institutes of Health Stroke Scale, *MT* mechanical thrombectomy, *mRS* modified Rankin Scale, *NL-ratio* neutrophil-lymphocyte ratio, *ASPECTS* Alberta Stroke Program Early CT Score, *CI* Confidence Interval

In follow-up imaging, all 10 ASPECTS regions were more commonly affected in patients who showed FR. In logistic regression, the ASPECTS regions internal capsule (OR (IC) 1.70 (1.05–2.74), *p* = 0.029) and insula (OR (IC) 1.68 (1.07–2.65), *p* = 0.024) as well as the cortical regions M2 (OR (IC) 1.88 (1.08–3.29), *p* = 0.025) and M5 (OR (IC) 2.71 (1.33–5.72), *p* = 0.007) were associated with FR (see Table 4).

**Table 4:** ASPECTS regions in follow-up imaging and association with FR

Affected ASPECTS region	Successful MT, not futile (277/532), *n* (%)	FR (255/532), *n* (%)	*p*-value, univariate	*p*-value, multivariable	Odds ratio (CI)
*Caudate nucleus*	65 (23)	96 (38)	**<** **0.001**	0.334	–
*Putamen*	100 (36)	96 (38)	**<** **0.001**	0.428	
*Internal capsule*	59 (21)	105 (41)	**<** **0.001**	**0.029**	1.698 (1.054–2.739)
*Insula*	101 (36)	165 (65)	**<** **0.001**	**0.024**	1.683 (1.069–2.649)
*Cortical regions*					
*M 1*	54 (19)	105 (41)	**<** **0.001**	0.371	–
*M 2*	48 (17)	120 (47)	**<** **0.001**	**0.025**	1.882 (1.083–3.291)
*M 3*	30 (11)	76 (30)	**<** **0.001**	0.422	–
*M 4*	21 (8)	65 (25)	**<** **0.001**	0.273	
*M 5*	17 (6)	79 (31)	**<** **0.001**	**0.007**	2.712 (1.333–5.719)
*M 6*	22 (8)	69 (27)	**<** **0.001**	0.173	–

## Discussion

Futile recanalization after mechanical thrombectomy in the anterior circulation is not uncommon and yet poorly understood despite all progress in modern stroke care [22]. Also, the definition of FR varies, but is majorly understood as absence of good clinical outcome measured with the NIHSS or the modified Rankin Scale. For this study it was defined as functional independence (mRS 0–2, 90 days after stroke onset) or mRS post-stroke > mRS pre-stroke. In this study with a large prospective single-center cohort and with a large amount of considered relevant covariates, we could define independent predictors for futile recanalization in anterior circulation ischemic stroke patients for two different scenarios. When implementing only variables that were known before MT: male sex, absence of thrombolysis before MT, pre-stroke mRS, NIHSS, age, the neutrophil-lymphocyte ratio in peripheral blood and lower ASPECTS score on admission were predictive for FR. The performance of the logistic regression model was good with an AUC of 0.78 but could still be improved to an AUC of 0.86 with adding predictive parameters that are only known after treatment initiation, namely failure of early neurological improvement, ASPECTS in follow-up imaging and certain levels of the Swieten score. On first sight the decreased influence of a higher Swieten score, which represents the degree of cerebral microangiopathic disease, in the final models seems contradictory. This might be due to certain cross-correlations between the predictors. Univariate regression modelling showed increased risk associated with higher Swieten scores. When analyzing the different ASPECTS regions in follow-up imaging, affection of the internal capsule, the insula or cortical regions M2 or M5 were predictive for FR.

Our results are in line with a smaller cohort study by van Horn et al. showing NIHSS, age and lower ASPECTS to be predictive for poor outcome after successful MT [23]. Interestingly, some variables with previously reported predictive properties for FR, like the cerebral collateral status were not predictive for FR in our study [24, 25]. The neutrophil-lymphocyte ratio (NLR) in peripheral blood samples was also shown to be predictive for poor outcome after stroke and MT in this study. Neutrophils and lymphocytes play major roles in the local inflammatory response of the brain following acute ischemic stroke. In this setting, neutrophils are taken as the motor of the inflammatory cascade while a neuroprotective ability is ascribed to lymphocytes [26]. Meanwhile, acute stroke might cause an immunosuppression state, which triggers lymphopenia. While the exact pathomechanism behind an elevated NLR and its relation to poor outcome after ischemic stroke is still unknown, it recently gained attention on account of its potential to become a cost-effective as well as easily available biomarker [27].

We previously showed in the same cohort that MT alone compared to MT with additional intravenous thrombolysis was associated with more frequent failure of early neurological improvement [28], and can now confirm this finding also for failure of long-term functional independence after successful MT. Our results are therefore adding evidence on the current debate of efficacy, safety and cost of thrombolysis in patients with LVO and direct access to MT in favor of bridging intravenous thrombolysis before MT [29, 30]. The male sex as predictive variable for FR in this study reflects sex-related differences in stroke epidemiology and etiology. So far, there is emerging evidence about existing sex-related difference in stroke epidemiology and outcome but the connecting factors, let alone probable pathomechanisms, remain largely unknown [31]. Interestingly, according to Rexrode et al. women are known to have a higher life-time risk of ischemic stroke. In a smaller cohort study, Ciardi et al. showed that women are older and have a longer delay to hospital admission but are equally likely to have a good clinical outcome after ischemic stroke and endovascular stroke treatment [32].Fifi et al. recently showed women to have a poorer outcome 90 days after stroke and MT [33]. In MR CLEAN women were more likely to die as well as have serious adverse events connected to MT, while a later meta-analysis (including MR CLEAN) did not reproduce these findings [1]. Our results, points to a disadvantage for the male sex, when it comes to poor outcome despite successful MT, which is partly contradictory to the abovementioned earlier studies favoring the male sex for more positive outcome.

The performance of our prediction model with variables known prior to the treatment decision was good but substantially improved further if parameters were included that were only obtainable after MT in the further clinical course of the patients, like ASPECTS on follow-up imaging (i.e. final infarct extension). Despite being (i) a severe complication, symptomatic intracranial hemorrhage (sICH) or being common (ii), like asymptomatic ICH after MT, both parameters were not selected in the second models, thereby limiting their predictive performance for FR in this cohort. While it is not surprising that also initially asymptomatic ICH does not influence the long-term disability after ischemic stroke and MT, the lacking predictive value of symptomatic ICH is rather atypical. This might be due to related associations of symptomatic ICH and other predictors regarding long-term disability after ischemic stroke and MT in this study. As mentioned above, we have previously shown that failure of early neurological improvement (fENI) after successful MT can be predicted by a variety of parameters. In this study, we were able to demonstrate that fENI itself was a strong independent factor for prediction of long-term functional independence after successful MT. Hence fENI might serve as an early clinical surrogate for rehabilitation planning and discussion of prognosis with family and relatives during acute stroke care.

### Limitations

All presented prediction models were built based on data from only a single center. Thus, external validation is still required for broad application. Affection of individual ASPECTS regions on follow-up imaging might further inform physicians on outcome prediction. Consistent to the association of affected basal ganglia (internal capsule), insula and cortical central region (ASPECTS region M2 and M5) with failure of early neurological improvement, these regions showed also to be associated with FR three months after stroke onset. However, this finding has to be seen in the light of the modified Rankin Scale potentially overestimating motor disabilities compared to other physical or psychological handicaps after ischemic stroke.

## Conclusion

This study revealed several predictors for missing functional independence despite full reperfusion of anterior circulation stroke patients that are available before mechanical thrombectomy and might inform physicians in individual cases on whether to treat a patient (potentially outside guidelines) or not. Outcome prediction is more precise if variables are included that are not available at the time physicians need to make a treatment decision. One of the strongest predictors of long-term functional independence after MT was shown to be failure of early neurological improvement.

## Supplementary Information

Details of the statistical analysis and calibration plots of the described prediction models

## Abbreviations

ASPECTS: Alberta stroke program early CT score
CV: Cross-validation
ENI: Early neurological improvement
fENI: Failure of early neurological improvement
HBC: Heidelberg bleeding classification
ICH: Intracranial hemorrhage
LVO: Large vessel occlusion
mRS: Modified Rankin Scale
MT: Mechanical thrombectomy
mTICI: Modified treatment in cerebral ischemia
NIHSS: National Institutes of Health Stroke Scale
NLR: Neutrophile-lymphocyte ratio
rtPA: Recombinant tissue plasminogen activator
