# Supplementary material for: Full Reperfusion Without Functional Independence After Mechanical Thrombectomy in the Anterior Circulation: Performance of Prediction Models Before Versus After Treatment Initiation
Source: Clin Neuroradiol. 2022 May 9;32(4):987–95. doi: 10.1007/s00062-022-01166-x (PMC9744692; doi:10.1007/s00062-022-01166-x)

# Supplemental Material

**Statistical Analysis – Model development**

The amount of missing values in the underlying dataset is 2.56%. Missing values were multiply imputed with 10 iterations. With respect to perform variable selection using cross-validation (CV), the 10 imputed data sets were merged by taking the mean for continuous and the mode of categorical variables. For the purpose of selecting variables with predictive impact on the primary endpoint (modified Rankin Scale (mRS) score of 3-6 three months after stroke onset or worse than baseline if the premorbid mRS was ≥ 3) regularized logistic regression using the elastic net penalty was applied on the merged data set using the glmnet package^16, 17^. The hyperparameters α (elastic net mixing parameter) and λ (shrinkage parameter) were tuned by a grid search and five-fold CV optimizing the area under curve (AUC) of the receiver operating characteristic (ROC) using the caret package^31^. Variable selection was then performed by fitting an elastic net logistic regression model with the optimized hyperparameters based on the entire merged data set to take all available information into account. Subsequently, multivariable regression modeling was conducted based on the ten imputed data sets consisting of all patients only incorporating the selected variables. In order to produce unbiased and finite parameter estimates, Firth's bias-reduced penalized-likelihood logistic regression was applied using the logistf package^32^. To obtain pooled confidence intervals of the respective ten parameter estimates after multiple imputation the combination of penalized likelihood profiles (CLIP) method was conducted^16^. The discriminatory performance of the final prediction model was assessed by calculating the AUC value based on the predicted risks for each patient during five-fold CV. The corresponding 95% confidence interval (CI) of the AUC value was calculated according to DeLong et al.^17^. The model’s calibration is assessed by calculating the calibration intercept and slope applying a flexible calibration curve using the CalibrationCurves package^33^. For the development of the two prediction models, the same methodical strategy was applied but a different set of variables was used as initial candidate predictors (variables available before and after MT, see Table 1).

# Figure I – Calibration plot of the Firth’s bias-reduced logistic regression model predicting futile recanalization before treatment initiation


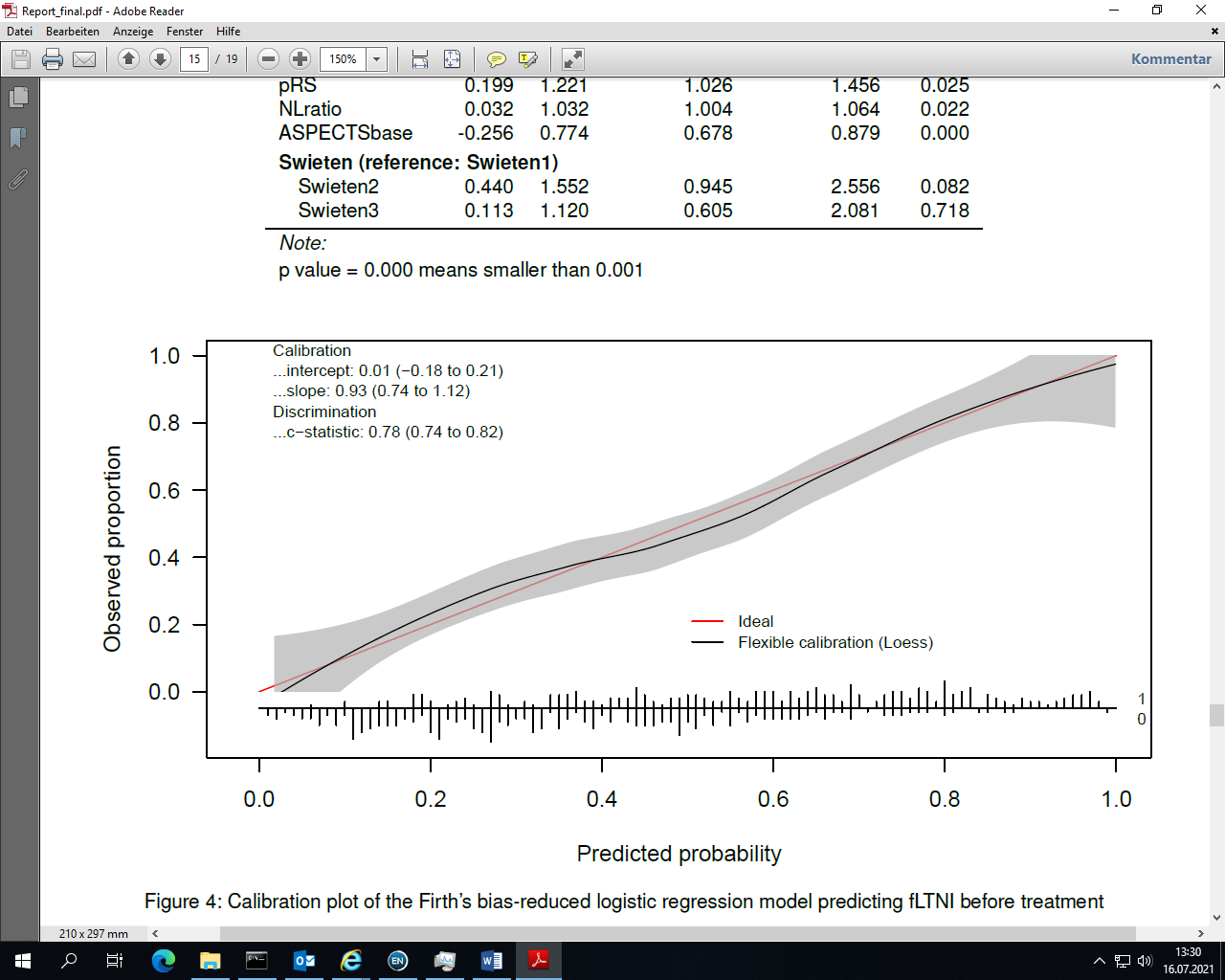


**Figure II – Calibration plot of the Firth’s bias-reduced logistic regression model predicting futile recanalization after treatment initiation**


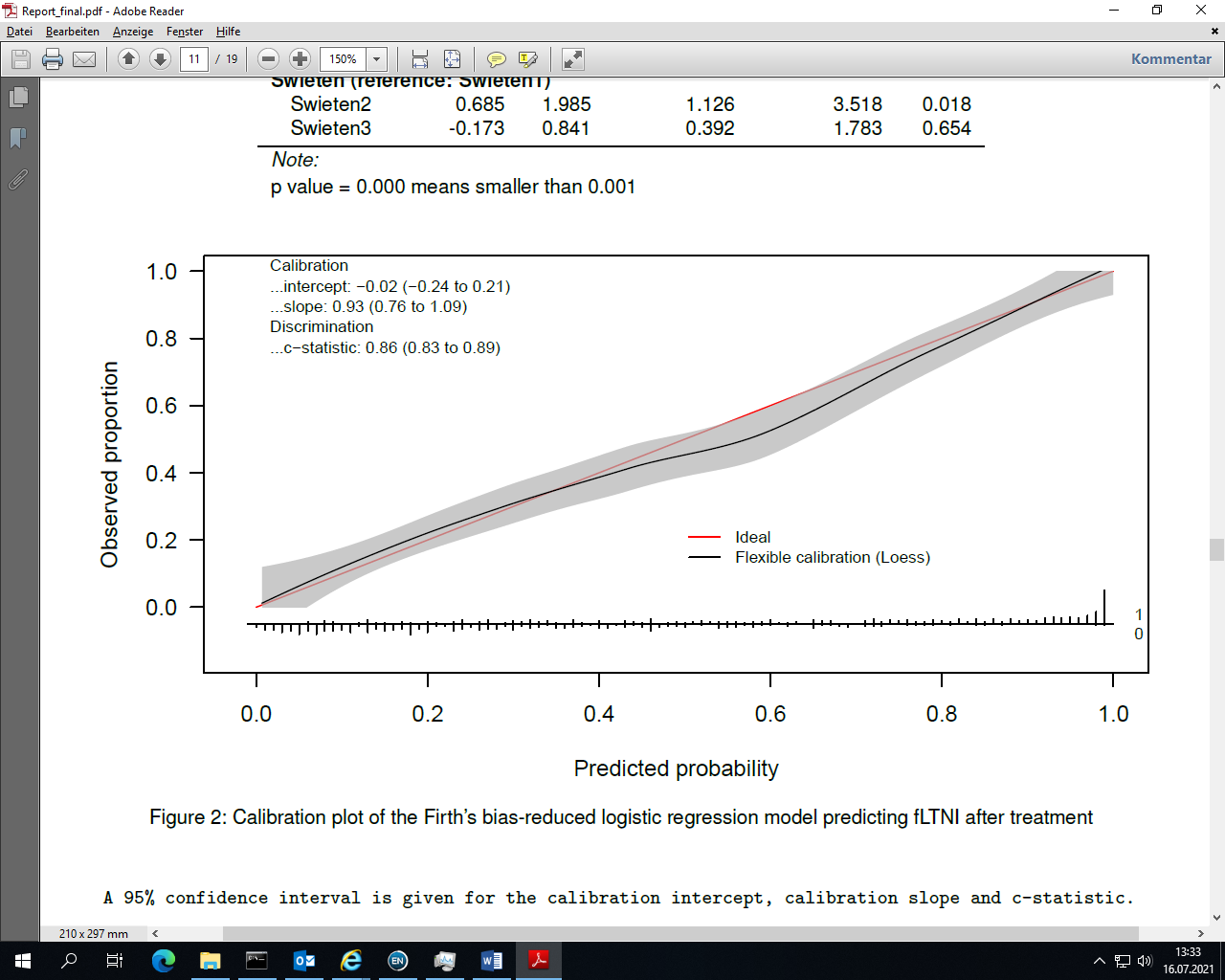

Supplement: Supplementary file 1 — Details of the statistical analysis and calibration plots of the described prediction models [file 62_2022_1166_MOESM1_ESM.docx]
